# A Longitudinal Investigation of Moral Injury Appraisals Amongst Treatment-Seeking Refugees

**DOI:** 10.3389/fpsyt.2018.00667

**Published:** 2018-12-18

**Authors:** Angela Nickerson, Joel Hoffman, Matthis Schick, Ulrich Schnyder, Richard A. Bryant, Naser Morina

**Affiliations:** ^1^School of Psychology, University of New South Wales, Sydney, NSW, Australia; ^2^Department of Consultation-Liason Psychiatry and Psychosomatic Medicine, University Hospital Zurich, University of Zurich, Zurich, Switzerland

**Keywords:** moral injury, refugees, posttraumatic stress disorder, depression, trauma

## Abstract

There is currently an unprecedented number of forcibly displaced people worldwide. Understanding psychological mechanisms that contribute to the mental health of refugees and asylum-seekers is important for informing the development of effective interventions for these populations. Moral injury appraisals represent an important potential cognitive mechanism that may contribute to psychological symptoms following exposure to persecution, war, and displacement. In the current study, we investigated the longitudinal association between moral injury appraisals related to one's own perceived transgressions (moral injury-self), others' perceived transgressions (moral injury-other), and PTSD and depression symptoms. Participants in this study were 134 refugees receiving treatment at two outpatient clinics in Switzerland who completed survey measures investigating these concepts. Of these, 71 were followed up 2 to 4 years later. Path analyses revealed that greater depression symptoms were associated with subsequent increases in moral injury-self appraisals (β = 0.25, SE = 0.08, 95% CI [0.11, 0.43], *p* = 0.002). In contrast, greater moral injury-self appraisals were associated with subsequent decreases in PTSD symptoms (β = −0.23, SE = 0.11, 95% CI = [−0.44, −0.31], *p* = 0.035). Findings suggest that different types of moral injury appraisals may be associated with differential psychological outcomes. These results have important potential implications for policy and treatment of refugees and asylum-seekers, highlighting the importance of targeting cognitive factors in the maintenance and treatment of psychological distress, and considering the post-migration context when working with refugees.

## Introduction

The number of people who have fled their homes as a result of war and persecution internationally is currently over 68 million (1). Compared to the general population in host countries, refugees report elevated rates of psychological disorders including posttraumatic stress disorder (PTSD) and depression (2, 3). The development of targeted interventions to reduce the psychological burden of refugees would be greatly aided by understanding the processes underlying psychopathology in these groups. To date, however, relatively little is known about the psychological mechanisms underpinning the mental health of refugees.

One psychological mechanism that has been consistently demonstrated to impact posttraumatic mental health both cross-sectionally and longitudinally is cognitive appraisals (4–7). Models of PTSD posit that the way in which the trauma survivor interprets their experiences, symptoms, and the broader context influences the development of symptoms in the aftermath of a potentially traumatic event [PTE; (8–10)]. The types of PTEs to which refugees are typically exposed (e.g., prolonged, repeated, human-instigated) are likely to engender cognitive change regarding the self and broader society, which may, in turn, contribute to psychopathology (11, 12). Despite a strong theoretical rationale, however, there is relatively little available evidence regarding cognitive appraisals that may arise from refugee experiences nor how these interact with mental health.

One framework that may be useful in conceptualizing the cognitive impact of the refugee experience is moral injury (13, 14). Moral injury can be defined as “the lasting psychological, biological, spiritual, behavioral, and social impact of …… bearing witness to acts that transgress deeply held moral beliefs and expectations” (15). This construct was initially developed to describe the effects of being exposed to moral transgressions in the context of combat (15); however, it is increasingly being applied to non-military settings (16–20). While moral injury is often conceptualized in terms of the nature of a specific event (e.g., killing in the context of warfare, betrayal by a superior officer) (15, 21), we posit that it can also be considered as a cognitive interpretation relating to refugee experiences, whereby the *appraisal* of an experience as morally injurious might give rise to psychological symptoms (13). This is broadly consistent with the construct of “moral pain,” proposed to describe the cognitive and emotional response to potentially morally injurious events (22). For example, in a refugee context, one individual being forced to leave behind a family member may consider this action a serious transgression of importan values; while to another, it may be perceived as a necessary step in moving one's immediate family to safety. Consistent with cognitive models of post-trauma mental health, these appraisals may then give rise to different psychological outcomes, with the former potentially contributing to psychological symptoms, and the latter to better post-trauma adaptation (8).

Research conducted in military settings has focused on two types of moral injury that may be especially relevant to refugees- that related to one's own actions (which we term moral injury-self) and that related to the actions of others (moral injury-other) (23, 24). Mixed findings have emerged regarding the association between these two types of moral injury and psychological outcomes in military samples. Currier et al. (24) found that both types of moral injury were associated with greater PTSD and depression symptoms. Although Bryan et al. (23) also found that moral injury-other was linked to higher levels of PTSD symptoms, only moral injury-self was associated with higher levels of hopelessness and anger. Held et al. (25) found that negative beliefs about others, negative beliefs about the self and self-blame mediated the association between moral injury-self (but not moral injury-other) and post-traumatic symptomatology in treatment-seeking veterans. Overall the differential association between different types of morally injurious events, appraisals, and psychological outcomes remains unclear.

To date, there have been two empirical investigations of moral injury appraisals in refugees. The first found that higher levels of moral injury-other were associated with greater PTSD and depression symptoms, explosive anger, and lower mental health-related quality of life in treatment-seeking refugees, over, and above the impact of PTE exposure, post-migration living difficulties (14). This study did not investigate moral injury-self. The second study investigated both moral injury-self and moral injury-other in a sample of 222 resettled refugees from diverse backgrounds (13). This study found that moral injury-self and moral injury-other were both predicted by greater trauma exposure, and associated with more severe anger and depression. While moral injury -other was associated with greater PTSD symptoms across all symptom clusters, moral injury -self was related to lower intrusive symptoms. Taken together, these findings indicate that that moral injury -self and moral injury -other may represent distinct constructs in a refugee context, and are differentially associated with psychological outcomes. To date, however, moral injury appraisals in refugees have only been investigated cross-sectionally. Without understanding how these types of moral injury appraisals relate to one another and psychological symptoms over time, it is difficult to ascertain the mechanistic role they may play in psychopathology in refugee groups.

The current study builds upon the Nickerson et al. study (14) to conduct the first longitudinal investigation of moral injury in refugees, with participants in this study being assessed 2 to 4 years after the first study. In addition, in this study both MI-other and MI-self were examined to elucidate their interrelationship as well as their relationship with important psychological outcomes. Understanding how moral injury appraisals might change over time, and their association to psychological symptoms, affords a unique opportunity to investigate the potentially mechanistic role of moral injury in the development and maintenance of psychological disorders.

In this study, we employed path analyses to investigate the association between moral injury appraisals and mental health over time. We hypothesized that:

(1) Greater exposure to PTEs and post-migration living difficulties would predict higher moral injury-self and moral injury-other appraisals. We based this prediction on the strong association between PTEs, post-migration living difficulties and psychological symptoms in the literature (26, 27), and the findings from Hoffman et al. (13) that greater trauma exposure was associated with higher moral injury-self and other appraisals.

(2) Higher moral injury-other appraisals at Time 1 would be associated with higher moral injury-self appraisals at Time 2, and vice versa. We based this prediction on findings from Hoffman et al. (13) that MI-self and MI-other were positively related cross-sectionally.

(3) Higher moral injury-other appraisals at Time 1 would be associated with greater PTSD and depression symptoms at Time 2. We based this prediction on findings from both Nickerson et al. (14) and Hoffman et al. (13) that MI-other was related to higher PTSD and depression cross-sectionally.

(4) Higher moral injury-self appraisals at Time 1 would be associated with greater depression (but not PTSD symptoms) at Time 2. We based this prediction on the finding from Hoffman et al. (13) that moral injury-self was associated with greater depression cross-sectionally, but not related to PTSD symptom sub-scales of avoidance, negative alterations in cognition and mood or hyperarousal.

## Materials and Methods

### Participants

Participants in this study were 134 refugees who, at the first time-point, were receiving psychological treatment at two outpatient clinics in Zurich and Bern, Switzerland. Exclusion criteria included current psychotic symptoms, severe dissociative symptoms, or active suicidality, which were assessed upon intake into the clinic by the treating psychiatrist or psychologist. At Time 1, participants in this study comprised 105 males (78.4%), with a mean age of 42.44 years (SD = 9.8%). Marital status of the sample was as follows: single *n* = 40 (29.9%), in a relationship/married *n* = 78 (58.2%) and divorced or widowed *n* = 16 (11.9%). The highest level of educational attainment was as follows: not completed primary school *n* = 17 (12.7%), completed primary school *n* = 43 (22.1%), completed high school *n* = 31 (23.1%), competed a Bachelor's Degree or technical college diploma *n* = 22 (23.9%) and completed a postgraduate degree *n* = 10 (7.5%). The employment status of the sample was as follows: engaged in full-time employment *n* = 9 (6.7%), engaged in part-time employment *n* = 17 (12.6%), unemployed *n* = 82 (61.2%) and retired or homemaker *n* = 23 (17.1%). At Time 1, participants had been in Switzerland for a mean of 9.01 years (SD = 6.7). Participants at Time 1 were from a number of ethnic backgrounds, including Turkey (*n* = 71, 53%, with N = 58, 43.3% being Kurdish), Iran (*n* = 15, 12%), Sri Lanka (*n* = 11, 8%), Bosnia (*n* = 6, 5%), Iraq (*n* = 6, 5%), Afghanistan (*n* = 5, 4%), and other (*n* = 20, 13%). At Time 1, participants had received treatment for a mean of 37.67 months (SD = 28.5). Inclusion criteria for this study comprised: (a) aged 18 years or older, and (b) speaking one of the study languages (German, Turkish, Arabic, Farsi, or Tamil).

Time 2 data collection commenced 2 years after Time 1, and 71 participants completed the second survey a mean of 2.81 (SD = 0.42) years after the Time 1. The distribution of ethnicities at Time 2 were as follows: Turkey (*n* = 42, 59.2%), Iran (*n* = 6, 8.5%), Sri Lanka (*n* = 6, 8.5%), Iraq (*n* = 4, 5.6%), and other (*n* = 13, 18.3%). Differences in the groups who completed the survey at Time 1 only, compared to those who completed the survey at Time 1 and Time 2 are presented in Table 1. Compared to those who completed only Time 1, participants who completed both time-points were significantly older [*t*_(132)_ = −3.13, *p* = 0.002], had lived in Switzerland longer [*t*_(132)_ = −2.65, *p* = 0.01] and included proportionally more males than females [*x*^2^_(1)_ = 4.24, *p* = 0.04], than participants who only completed Time 1.

**Table 1:** Differences between participants who completed time 1 and time 2 survey measures, and participants who completed only time 1 survey measures (*t*-tests).

**Variable**	**Completed T1 & T2** ***M*(*SD*)**	**Completed T1 only** ***M*(*SD*)**	***t***	***df***	***p***	***95% CI***
Age	44.83(8.96) [*n* = 71]	39.75(10.12) [*n* = 63]	3.08	132	0.002	1.82, 8.35
PTE exposure	12.49(4.34) [*n* = 71]	11.97(4.79) [*n* = 63]	0.67	132	0.507	−1.04, 2.09
LDC	10.03(4.26) [*n* = 71]	9.48(4.06) [*n* = 63]	0.77	132	0.445	−0.87, 1.98
Length of time in [edited out for blind review]	10.41(6.70) [*n* = 71]	7.44(5.96) [*n* = 63]	2.65	132	0.010	−0.73, 5.20
PTSD	1.73(0.68) [*n* = 64]	1.64(0.69) [*n* = 55]	0.77	132	0.442	−0.14,0.33
Depression	2.73(0.58) [*n* = 68]	2.71(0.66) [*n* = 59]	0.22	132	0.687	−0.19,0.24
Anger	1.39(0.75) [*n* = 68]	1.16(0.66) [*n* = 59]	1.87	131	0.064	−0.01,0.47
MI–Self	1.80(1.02) [*n* = 68]	1.70(0.99) [*n* = 59]	0.74	125	0.464	−0.18,0.22
MI–Other	1.07(0.74) [*n* = 69]	0.86(0.70) [*n* = 61]	1.67	128	0.097	−0.13,0.04
Gender (male, female)	(*n =* 61, *n =* 10)	(*n =* 45, *n =* 18)	***X**^**2**^ = 4.24*	1	0.400

*t, t-statistic; df, degrees of freedom; p, significance level; 95%CI, 95% confidence interval; X^2^, chi-squared statistic; MI-Other, moral injury appraisals of violations by others; MI-Self, moral injury appraisals of violations by oneself; PTE, potentially traumatic events; LDC, post-migration living difficulties checklist; PTSD, posttraumatic stress disorder*.

### Measures

Gold-standard translation and back-translation procedures were implemented for measures in this study (28). Measures were first translated from English into the study languages (German, Turkish, Arabic, Farsi, or Tamil) by accredited translators, and were then translated back into English by translators who had not seen the original versions of the questionnaires. Discrepancies, in the form of minor language inconsistencies, were rectified jointly by the research team and bilingual individuals with experience working with mental health-related information.

### Exposure to Potentially Traumatic Events

We indexed exposure to PTEs using a measure developed for this study. The trauma event lists of two standardized questionnaires, the Harvard Trauma Questionnaire (29) and the Posttraumatic Diagnostic Scale (30) were amalgamated, and the final scale comprised 23 items which indexed exposure to PTEs typically experienced by refugees. Example events included witnessing the death of loved ones, lack of food and water, and torture. PTE exposure was indexed by a count of the number of types of traumatic events experienced by each participant.

### Post-migration Stressors

Post-migration stressors were assessed using the Post-Migration Living Difficulties Checklist (31, 32), which was adapted to the Swiss context. This scale comprised 17 items detailing common difficulties experienced by refugees in their host society (e.g., language difficulties, financial difficulties). Participants indicated the extent to which each of these stressors had been a problem over the past 12 months on a five point scale (0 = *not a problem* to 4 = *a very serious problem*). Items that scored 2 (*a moderately serious problem*) or more were considered significant stressors, and a total count of living difficulties was derived.

### PTSD Symptoms

We measured PTSD symptoms using the Posttraumatic Diagnostic Scale (30). As data collection commenced prior to the release of the updated version of this scale, four items were added to encompass new DSM-5 criteria for PTSD and one item from the obsolete DSM-IV criteria removed, resulting in a 20-item scale (33). Participants rated items on a 4-point scale (0 = *not at all/ only once* to 3 = five *or more times a week/ almost always*). We used a total sum score to represent PTSD symptom severity. This scale has been used to assess PTSD in refugees (34–36), and has strong psychometric properties (37). The internal consistency for this scale was α = 0.94.

### Depression Symptoms

Depression was measured using the 15-item depression subscale of the Hopkins Symptom Checklist (38). Items were measured on a 4-point scale (1 = *not at all* to 4 = *extremely*), and a total sum score used to represent depression symptom severity. The HSCL has been used on numerous occasions with refugees (36, 39), and has strong psychometric properties (38). Internal consistency for this scale was α = 0.89.

### Moral Injury Appraisals in Refugees

A 6-item scale indexing moral injury was developed for this study (14). This scale comprised two subscales, namely moral injury relating to one's own actions (MI-Self) and moral injury relating to others' actions (MI-Other). All items were rated on a 4-point scale (0 = *not at all*, 3 = *very much*). For each subscale, a mean of items was taken to represent the subscale score. The *MI-Self* subscale comprised two items relating to the individual being troubled because of actions they took or failed to take that transgressed their morals. As Cronbach's alpha provides biased estimates for reliability for two-item scales, a Spearman-Brown coefficient was calculated for this scale, ρ = 0.71 (40). The *MI-Other* subscale comprised four items relating to the individual being troubled by the actions of others that transgressed their morals. The internal consistency for this scale was α = 0.77.

### Procedure

Ethics approval was received for this study from the Ethics Committees of the Cantons of Zurich and Bern. All eligible individuals who were receiving treatment at the Zurich and Bern clinics were invited to take part in the study by their treating therapist or a study team member. Participants were informed that their decision to participate or not would have no impact on the treatment they received at these clinics. Participants attended a 1 to 2 h long research session where they first provided written informed consent. Participants completed questionnaires in their own language using a therapist-assisted computer-based assessment tool (MAPSS) (41). Self-report questionnaires were presented on a tablet in written and auditory form. Psychiatrists, clinical psychologists or masters-level students of clinical psychology supervised the research session. Interpreters were used for sessions where required. Participants were reimbursed CHF 40 (approx. USD 40) for participation. Two to four years after completion of the first session, participants were contacted by the study team to invite them to complete the second survey. The same procedure was followed for the second session.

### Data Analysis

Descriptive statistics were generated using SPSS Version 23.0. Path analysis, conducted in Mplus version 7.4, was used to investigate the relationship between demographics, refugee experiences, moral injury subscales, and PTSD, and depression symptoms at Time 1 and Time 2. There was 47.0% attrition at Time 2 due to participants being uncontactable (*n* = 44) or declining to participate (*n* = 19). A full information maximum likelihood (FIML) estimator was implemented. This estimator is ideal for models where there is substantial missing data as it uses other variables in the model to account for missing values (42). Variables on which individuals who dropped out of the study differed from those who completed both time-points were included in the model to create unbiased parameter estimates. The model was initially specified as follows: Step 1: age, gender, length of time in Switzerland, trauma exposure and post-migration living difficulties (all measured at Time 1); Step 2: (MI-self, MI-other, PTSD symptoms, and depression symptoms (measured at Time 1); Step 3: MI-self, MI-other, PTSD symptoms, and depression symptoms. All variables were permitted to influence variables at the subsequent step. Residual error variance was allowed to covary between variables entered at the same step.

Next, model trimming was employed and paths with *p* < 0.10 were removed from the model. Indirect effects were tested using bootstrapping (500 draws), which yields 95% confidence intervals. Model fit criteria included a non-significant chi-square test χ^2^ test, a root mean square error of approximation (RMSEA) <0.06, Comparative Fit Index (CFI) ≥0.90, Tucker-Lewis Index (TLI) ≥0.90, and standardized root mean square residual (SRMR) <0.05 (43).

## Results

### Exposure to Potentially Traumatic Events and Post-migration Living Difficulties

Participants had experienced a mean of 12.25 types of PTEs (*SD* = 4.54). The most frequent types of events experienced were torture (85.1%), imprisonment (76.9%), and enforced isolation from others (76.9%). Participants had also experienced a mean of 9.77 types of living difficulties (*SD* = 4.16). The most frequent types of living difficulties experienced were loneliness, boredom, or isolation (84.3%), worries about family back home (80.6%), and being unable to return to their home country in an emergency (75.4%).

### Path Analysis

**Figure 1:**
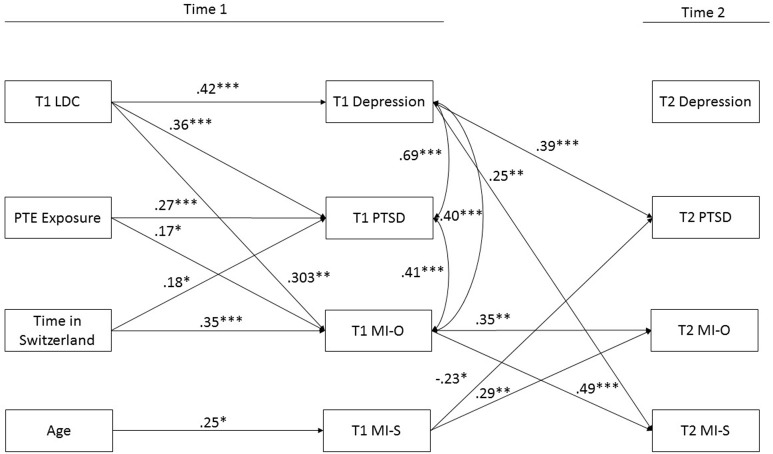
Longitudinal association between refugee experiences, moral injury appraisals and psychological symptoms in traumatized refugees. LDC, Postmigration living difficulties; PTE, Potentially Traumatic Events; T1, Time 1; T2, Time 2; MI-O, Moral injury Other; MI- S, Moral Injury Self. ^*^p < 0.05, ^**^p < 0.01, ^***^p < 0.001.

The initial model in which all paths were specified yielded adequate fit $\chi_{\left(20\right)}^{2}$ = 23.90, *p* = 0.247, RMSEA = 0.04 (95% CI = 0.00 to 0.09), CFI = 0.99, TLI = 0.95, SRMR = 0.05. After the removal of paths with *p* < 0.10, good model fit was retained $\chi_{\left(31\right)}^{2}$ = 29.63, *p* = 0.537, RMSEA = 0.00 (95% CI = 0.00 to 0.06), CFI = 1.00, TLI = 1.01, SRMR = 0.06. Standardized parameter estimates of direct effects are presented in Figure 1 and Table 2. Standardized indirect effects are presented in Table 3.

**Table 2:** Standardized coefficients for direct effects in path model.

	**β**	***SE***	***95% CI***	***p***
**ON AGE**				
T1 MI-Self	0.248	0.123	(0.004, 0.486)	<0.044
**ON TIME IN SWITZERLAND**				
T1 Depression	0.163	0.084	(0.018, 0.338)	0.053
T1 PTSD	0.184	0.078	(0.035, 0.337)	0.018
T1 MI-Other	0.349	0.074	(0.194, 0.488)	<0.001
**ON PTE EXPOSURE**				
T1 Depression	0.157	0.089	(−0.008, 0.325)	0.076
T1 PTSD	0.269	0.086	(0.098, 0.430)	<0.001
T1 MI–Other	0.166	0.079	(0.002, 0.318)	0.036
**ON T1 LDC**				
T1 Depression	0.419	0.081	(0.268, 0.571)	<0.001
T1 PTSD	0.361	0.086	(0.183,0.518)	<0.001
T1 MI–Other	0.303	0.096	(0.099, 0.479)	0.002
**ON T1 DEPRESSION**				
T2 Depression	0.210	0.117	(−0.036, 0.422)	0.072
T2 PTSD	0.391	0.102	(0.183, 0.605)	<0.001
T2 MI–Self	0.252	0.082	(0.110, 0.425)	0.002
**ON T1 MI**–**OTHER**				
T2 MI–Other	0.352	0.108	(0.101, 0.542)	0.001
T2 MI–Self	0.491	0.071	(0.327, 0.613)	<0.001
**ON T1 MI**–**SELF**				
T2 PTSD	−0.230	0.109	(−0.440, −0.031)	0.035
T2 MI-Other	0.286	0.109	(0.079, 0.495)	0.009

*β, standardized Beta coefficients; SE, standard error; 95% CI, 95% confidence interval; p, significance; PTE, potentially traumatic events; LDC, post–migration living difficulties; MI-Other, moral injury appraisals of violations by others; MI-Self, moral injury appraisals of violations by oneself; PTSD, posttraumatic stress disorder*.

**Table 3:** Standardized indirect effects for path model.

	***B***	***SE***	***95%CI***	***p***
**PTE EXPOSURE TO T2 PTSD**				
Via T1 Depression	0.061	0.039	(−0.001, 0.154)	0.118
**PTE EXPOSURE TO T2 DEPRESSION**				
Via T1 Depression	0.033	0.027	(−0.002, 0.100)	0.217
**PTE EXPOSURE TO T2 MI**–**OTHER**				
Via T1 MI-Other	0.058	0.033	(0.011, 0.135)	0.078
**PTE EXPOSURE TO T2 MI-SELF**				
Total indirect	0.121	0.057	(0.002, 0.230)	0.034
Via T1 MI-Other	0.081	0.041	(−0.002, 0.166)	0.049
Via T1 Depression	0.040	0.026	(0.000, 0.101)	0.130
**T1 LDC TO T2 PTSD**				
Via T1 Depression	0.164	0.055	(0.071, 0.305)	0.003
**T1 LDC TO T2 DEPRESSION**				
Via T1 Depression	0.088	0.054	(−0.005, 0.213)	0.104
**T1 LDC TO T2 MI-OTHER**				
Via T1 MI-Other	0.106	0.043	(0.029, 0.194)	0.013
**T1 LDC TO T2 MI-SELF**				
Total indirect	0.254	0.067	(0.117, 0.372)	<0.001
Via T1 MI-Other	0.149	0.053	(0.054, 0.258)	0.005
Via T1 Depression	0.106	0.043	(0.042, 0.207)	0.013

*β, standardized Beta coefficients; SE, standard error; 95% CI, 95% confidence interval; p, significance; PTE, potentially traumatic events; MI-Other, moral injury appraisals of violations by others; MI-Self, moral injury appraisals of violations by oneself; LDC, living difficulties checklist; PTSD, posttraumatic stress disorder*.

### Hypothesis 1: Association Between Refugee Experiences and MI Appraisals

Greater exposure to PTEs and living difficulties at Time 1 significantly predicted higher MI-Other but not MI-Self appraisals at Time 2.

### Hypothesis 2: Association Between MI Appraisals at Time 1 and Time 2

Higher MI-Other appraisals at Time 1 were associated with higher MI-Self appraisals at Time 2. Higher MI-Self appraisals at Time 1 were associated with higher MI-Other appraisals at Time 2.

### Hypothesis 3: Association Between MI-Other Appraisals at Time 1 and Psychological Symptoms at Time 2

Contrary to our hypotheses, MI-Other appraisals at Time 1 were not associated with psychological symptoms at Time 2.

### Hypothesis 4: Association Between MI-Self Appraisals at Time 1 and Psychological Symptoms at Time 2

Higher MI-Self appraisals at Time 1 were associated with lower PTSD symptoms at Time 2. Notably, higher depression symptoms at Time 1 were associated with greater MI-Self appraisals at Time 2.

### Indirect Effects

There was a significant indirect effect of trauma on MI-Self appraisals at Time 2 via MI-Other appraisals at Time 1. There was a significant indirect effect of living difficulties on PTSD at Time 2 via depression symptoms at Time 1. There was a significant indirect effect of living difficulties on MI-Other appraisals at Time 2 via MI-Other appraisals at Time 1. There were significant indirect effects of living difficulties on MI-Self appraisals on Time 2 via MI-Other appraisals at Time 1, and depression symptoms at Time 1.

## Discussion

To our knowledge, this study represents the first longitudinal investigation of moral injury appraisals amongst refugees. The key finding from this study was that moral injury-other and moral injury-self appraisals were differentially associated with psychological outcomes in treatment-seeking refugees. Specifically, we found that depression at baseline predicted greater moral injury-self appraisals at Time 2, and moral injury-self appraisals at Time 1 predicted *lower* PTSD symptoms at Time 2. These findings suggest that higher depression symptoms may give rise to increased appraisals that the individual has transgressed their own morals. This is consistent with cognitive models of depression that highlight the powerful role of negative cognitions about the self in this disorder (44, 45). Our finding, that moral injury appraisals at Time 1 did not predict depression at Time 2, is contrary to traditional cognitive models of depression which have posited that maladaptive cognitions contribute to the development of psychological symptoms (44, 46). Other theoretical and empirical accounts, however, have suggested that the relationship between depression and negative cognitions is bidirectional (47), or that negative cognitions arise from, rather than precede, depression symptoms (48, 49). Accordingly, theorists have posited a reciprocal relationship between negative cognitions and depression, in which a cycle of self-recrimination, rumination, and depressed mood prolongs, and increases symptoms (50–52). Consistent with this, in a trial testing prolonged exposure therapy for PTSD, changes in depression symptoms preceded changes in self-blame cognitions (53). In this study, participants with high levels of depression may have engaged in rumination regarding their previous actions, thus intensifying their distress about perceived moral transgressions. It is important to note, however, that participants in this study were treatment-seeking, and thus these processes may have been influenced by treatment strategies and/or symptom reduction. Further research is required to elucidate the relationship between depressed mood, moral injury-self appraisals, and other cognitive processes such as rumination.

Our results revealed that higher levels of moral injury-self cognitions were associated with subsequent decreases in PTSD symptoms in this sample of treatment-seeking refugees. This is partly consistent with the findings from Hoffman et al. (13) that higher levels of moral injury-self cognitions were associated with lower intrusive (but not avoidance, cognitions and mood and hyperarousal symptoms). This is in contrast to cross-sectional research with military samples indicating that moral injury related to one's own actions is linked to higher PTSD symptoms (15, 21), although it should be noted that Bryan et al. (23) found that moral injury-self was not associated with PTSD in a military sample. While cognitive models of PTSD posit that higher levels of negative trauma-related appraisals give rise to more severe PTSD symptoms (8–10), evidence regarding the relationship between PTSD and negative appraisals of one's own actions is mixed. Studies conducted with survivors of various types of trauma have documented both a positive relationship between self-blame and PTSD symptoms (54–56), and no relationship between these variables (57–59). Further, consistent with the current findings, Startup et al. (60) found that higher levels of self-blame were associated with lower PTSD symptomatology in survivors of various types of traumatic events. The present findings can be interpreted in multiple ways. Firstly, as our sample were treatment-seeking, it may be that the observed reduction in PTSD symptom severity between Time 1 and 2 was related to psychotherapy received during this period. For example, if participants reported greater cognitions related to moral injury-self at Time 1, this may have led the therapist to focus the treatment on these appraisals, potentially producing a flow-on effect that resulted in reduced PTSD symptoms at Time 2. These findings should be replicated in a non-treatment seeking sample to determine whether these results represent an artifact of treatment. Second, it is possible that these findings point to a specific etiological pathway for the development and maintenance of PTSD. Models of PTSD highlight the protective role of perceived control during the traumatic event against the development of PTSD symptoms (8, 61, 62). Accordingly, in a study with accidental injury survivors, greater perceived responsibility for a traumatic event was associated with lower PTSD symptoms 6 months later (63). It is possible that, in this study, the appraisal that one's actions were transgressive was related to the perception that one could have acted differently during the event. This may have led to greater perceived control during the situation, and lower resulting levels of PTSD symptoms. Alternatively, the appraisal that one has acted against one's moral code may lead the traumatic memory to be experienced in a manner that is qualitatively distinctive. For example, individuals with higher levels of moral injury-self appraisals may experience lower levels of avoidance due to intentionally engaging with the traumatic memory. This may lead to the memory being processed differently, contributing to a unique symptom presentation. These explanations are highly speculative, however, and further longitudinal and experimental work is required to replicate this finding, and determine the mechanism by which it operates.

Contrary to our hypothesis, we found that moral injury-other appraisals did not predict subsequent psychological symptoms, despite being associated with PTSD and depression at baseline in both this study and our previous investigations (13, 14). These results suggest that the extent to which an individual perceives that they transgressed their own moral code may have a stronger association with PTSD and depression over time than the perception that the individual was transgressed against. One possible explanation for this result is that, while the appraisal that others have violated one's moral code is associated with high levels of concurrent symptomatology, this relationship may weaken over time. Another possibility is that moral injury-other appraisals are associated with differential reactions in the long-term, for example, higher levels of humiliation, hostility or mistrust of others. This is supported by findings from a longitudinal study conducted with survivors of persecution and war in Timor-Leste, which indicated that a sense of injustice relating to past events strongly predicted explosive anger reactions (in addition to PTSD and general psychological distress) (64). Further, Stein et al. (65) found that experiencing morally injurious events perpetrated by others was associated with feelings of humiliation in active duty service members. More research is required to determine the longitudinal psychological and behavioral effects of perceived moral transgressions enacted by others in refugees.

Consistent with our hypotheses, we found that moral injury-other, and moral injury-self were positively associated over time. Accordingly, the extent to which an individual perceives that they has transgressed and been transgressed against appear to be related, and contribute to the strengthening of these beliefs over time. This is consistent with the findings of Hoffman et al. (13), and research indicating that negative cognitions relating to traumatic events cluster together (66–68).

Also in accordance with our hypothesis, we found that greater moral injury-other appraisals were associated with greater exposure to PTEs and post-migration living difficulties. These findings are consistent with a large body of literature which shows a dose-response relationship between PTEs and post-migration living difficulties and negative mental health outcomes in refugees (26, 27, 69). This finding suggests that the more types of traumatic events to which an individual is exposed, the greater they sense of having been transgressed against. This is consistent with results suggesting that greater exposure to trauma was associated with greater perceptions of injustice in survivors of persecution and war (64). The positive association between exposure to post-migration living difficulties and moral injury-other appraisals is notable, as it provides preliminary evidence to suggest that moral injury appraisals may be associated with experiences beyond those that are traumatic in nature. This may be especially relevant to the case of refugees. For example, a refugee may, upon fleeing his or her country, hope that they will be warmly received into the host society, is able to obtain permanent residency, find employment, and support they family financially. If, for instance, the individual is subject to discrimination, temporary visa status, and is unable to get qualifications recognized, they may feel that the host society or representative government has acted in a way that transgresses his or her moral code, giving rise to moral injury-self appraisals. Further research is required to elucidate this finding. However, these results indicate that it may be important to look beyond traditionally conceptualized traumatic events when considering moral injury in refugees.

## Limitations

The current study was subject to several limitations. First and foremost, the measure used to index moral injury was preliminary in that it has not been validated with other populations. The two-item scale representing MI-Self was also limited in the extent to which it comprehensively captured MI-self appraisals, and the use of a two-item measure is not optimal from a statistical perspective at its reliability cannot be ascertained. Future research focusing on the development of a psychometrically valid measure of moral injury appraisals relating to refugees would be useful. Second, our sample size and, in particular, the number of individuals followed-up from the initial time-point, was small. This may have limited our statistical power in uncovering relationships between variables. Third, participants in this study were treatment-seeking. While this provides an ecologically-valid sample of individuals likely to experience psychological distress arising from moral injury, it is not possible to determine whether the relationships reported in this study were due to naturalistic change over time, as they were likely also influenced by therapeutic processes. Future longitudinal investigation of these variables in a community sample would be valuable. Fourth, there were a number of potential confounding factors that were not indexed in the current study, including the use of psychotropic medication which may have affected psychological symptoms, the varying length of time between baseline assessment and follow-up, which may have influenced results, and the type and frequency of psychological interventions received by participants during the follow-up period. Fifth, due to the small sample size it was not possible to evaluate the psychometric properties of each scale for each language group. Finally, there were a number of constructs of interest that were not investigated in this study. Examining the extent to which psychological processes such as rumination are related to moral injury, and potential outcomes beyond psychological symptoms (i.e., trust, hostility, humiliation) represents a future research direction.

## Clinical and Policy Implications

Results from this study may have important treatment and policy implications. The finding that moral injury-self appraisals were negatively associated with subsequent PTSD symptoms, and positively associated with prior depression symptoms raises important clinical considerations. It may be that these appraisals do not respond optimally to front-line exposure-based interventions for PTSD. This is consistent with findings from a clinical trial indicating that self-blame cognitions show less change during trauma-focused treatments than other cognitions (70), although another study found that reductions in depression symptoms in prolonged exposure therapy preceded self-blame cognitions (53). Nevertheless, moral injury-self appraisals may be more responsive to cognitive-based interventions for depression or PTSD, or specific treatments for moral injury adapted for refugees [i.e., Adaptive Disclosure (71, 72)]. Given the longitudinal association between MI-self appraisals and depression symptoms in this study, it would be useful to investigate whether specific treatment strategies are more effective in reducing moral injury-self and moral injury-other appraisals. From a policy perspective, it is important to note the association between post-migration living difficulties and moral injury-other appraisals in this study. There is a growing body of evidence highlighting the pivotal role of post-migration stressors in influencing outcomes in resettled refugees [for a review, see Li et al. (26)]. The finding that greater exposure to these stressors is associated with a higher perception of having been transgressed against suggests that it is important for host governments to develop supportive policies and provide adequate resources for refugees upon settlement to promote better adaptation to the new country and positive social outcomes.

## Conclusions

The current study represents, to our knowledge, the first longitudinal investigation of two types of moral injury appraisals (namely moral injury related to one's own and others' transgressions) in refugees. Findings indicated that these two types of appraisals are distinctive in terms of their association to refugee experiences, and to psychological outcomes over time. This research provides preliminary evidence that moral injury may be a useful framework for conceptualizing the cognitive experience of individuals who have been exposed to trauma, persecution, displacement and post-migration stress. Further research is required to elucidate the association between moral injury appraisals and psychological, social, and behavioral outcomes amongst refugees.

## Ethics Statement

The study was approved by the Ethics Committee of the Cantons of Zurich (Project Nr. KEK-ZH-Nr. 2011–0495) and Bern (KEK-BE_Nr. 152/12), Switzerland, and was conducted in compliance with the Code of Ethics of the World Medical Association (Declaration of Helsinki). All participants provided written informed consent prior to study participation.

## Author Contributions

AN, MS, US, and RB were involved in the conception of the study, the interpretation of the data and the drafting, and revision of the manuscript. JH was involved in the interpretation of the data and drafting and revision of the manuscript. NM was involved in the conception of the study, data collection, analysis, and interpretation of the data, and the drafting of the manuscript. All authors read and approved the final manuscript.

### Conflict of Interest Statement

The authors declare that the research was conducted in the absence of any commercial or financial relationships that could be construed as a potential conflict of interest.
